# Causal associations of hyperthyroidism with prostate cancer, colon cancer, and leukemia: a Mendelian randomization study

**DOI:** 10.3389/fendo.2023.1162224

**Published:** 2023-05-18

**Authors:** Feipeng Xu, Zhenxin Chen

**Affiliations:** ^1^ Department of Endocrinology, The First Hospital of Putian City, Putian, Fujian, China; ^2^ Department of Endocrinology, Teaching Hospital, The First Hospital of Putian, Fujian Medical University, Putian, Fujian, China

**Keywords:** Mendelian randomization, hyperthyroidism, prostate cancer, colon cancer, leukemia, genome-wide association studies

## Abstract

**Background:**

Observational studies have shown that hyperthyroidism may increase the risk of cancer, but their causal effects and direction are unclear. We conducted a two-sample Mendelian randomization (MR) study to explore the associations between genetic predisposition to hyperthyroidism and nine common types of cancer, including prostate, lung, breast, colon, leukemia, brain, skin, bladder, and esophagus cancer.

**Methods:**

We obtained summary statistics of hyperthyroidism and nine types of cancers from genome-wide association studies (GWAS). MR analysis is performed to investigate the potential causal relationship between hyperthyroidism and cancers. The inverse variance weighted (IVW) as the primary method was carried out. The robustness of the results was evaluated by sensitivity analysis.

**Results:**

Genetically predicted hyperthyroidism was associated with a declining risk of occurrence of prostate cancer (odds ratio (OR)_IVW_= 0.859, P= 0.0004; OR _MR-Egger_=0.828, P= 0.03; OR _weighted median_= 0.827, P=0.0009). Additionally, there was a significant association between genetically predicted hyperthyroidism and colon cancer (OR _IVW_= 1.13, P= 0.011; OR _MR-Egger_= 1.31, P= 0.004; OR _weighted median_= 1.18, P= 0.0009). Hyperthyroidism was also suggestively correlated with a higher risk of leukemia based on the result of IVW and weighted median (OR _IVW_= 1.05, P= 0.01; OR _weighted median_= 1.08, P= 0.001). Results from a two-sample MR analysis suggested that hyperthyroidism was not associated with the risk of lung cancer, breast cancer, brain cancer, skin cancer, bladder cancer, and esophageal cancer.

**Conclusion:**

Our study provides evidence of a causal relationship between hyperthyroidism and the risk of prostate cancer, rectal cancer, and leukemia. Further research is needed to clarify the associations between hyperthyroidism and other cancers.

## 1 Introduction

Hyperthyroidism is a pathological disease caused by thyroid synthesis and secretion of excessive thyroid hormones (THs), characterized by normal or high thyroid radioactive iodine uptake (1). THs are known for their roles in metabolism, growth, and development (2, 3). Due to the critical role of thyroid hormones in cell proliferation and differentiation, some studies consider hyperthyroidism a potential and preventable risk factor for cancer (4, 5).

A growing number of studies have revealed the tumor-promoting effect of THs (4, 6, 7). THs specifically act on the membrane and nuclear receptors, leading to the activation of a variety of carcinogenic pathways, thereby activating cell growth, inhibiting apoptosis, and stimulating angiogenesis. For example, T3 can activate the carcinogenic phosphatidylinositol-3 kinase (PI3K) pathway through thyroid hormone receptor (TRs), resulting in target gene transcription and overexpression of hypoxia-inducible factor-1a (HIF-1 α) α subunit (8). It is a medium adapted to hypoxia and angiogenesis and is critical in tumor cell growth, invasion, and metastasis (9). Furthermore, similar results have been observed in some observational studies. For instance, A prospective cohort study including more than 10,000 participants by Khan et al. showed that higher free T4 (FT4) levels were associated with an overall higher risk of solid cancer (adjusted HR 1.42); The association was somewhat weakened, though by excluding patients who were taking thyroid-influencing drugs (10). Simultaneously, Yeh et al. conducted a large prospective study to explore the association between hyperthyroidism and cancer. They found that hyperthyroidism was associated with an increased cancer risk (adjusted HR 1.20) (11). And the risk of cancer was positively correlated with the duration of hyperthyroidism. However, a significant limitation of the available evidence is that the results of observational studies are susceptible to residual confounders and reverse causality; this impedes causal inference of the relationship between hyperthyroidism and cancer risk.

Mendel randomization (MR) has gradually been used in epidemiology in recent years to infer causality between exposure and disease outcomes based on genetic variation and Single-nucleotide polymorphism (SNPs) (12). In MR, the causal inference of exposure-outcome associations is explored using phenotype-related genetic variants as instrumental variables (13–15). Genetic variation meets the rule that alleles are randomly separated from parents to offspring and is determined by genetic variation at the time of conception. Therefore, compared with traditional observational studies, MR is not easily affected by population confounding factors (14). In this study, we performed a two-sample MR obtaining data from summary statistics from large-scale genome-wide association studies (GWAS) of hyperthyroidism and nine types of cancers to explore the causal effect of hyperthyroidism on the risk of nine cancers carcinogenesis.

## 2 Methods

The data used in this study came from published studies or GWAS summaries, which were publicly available. No additional ethical approval was required.

### 2.1 Genetic variants associated with hyperthyroidism

**Figure 1:**
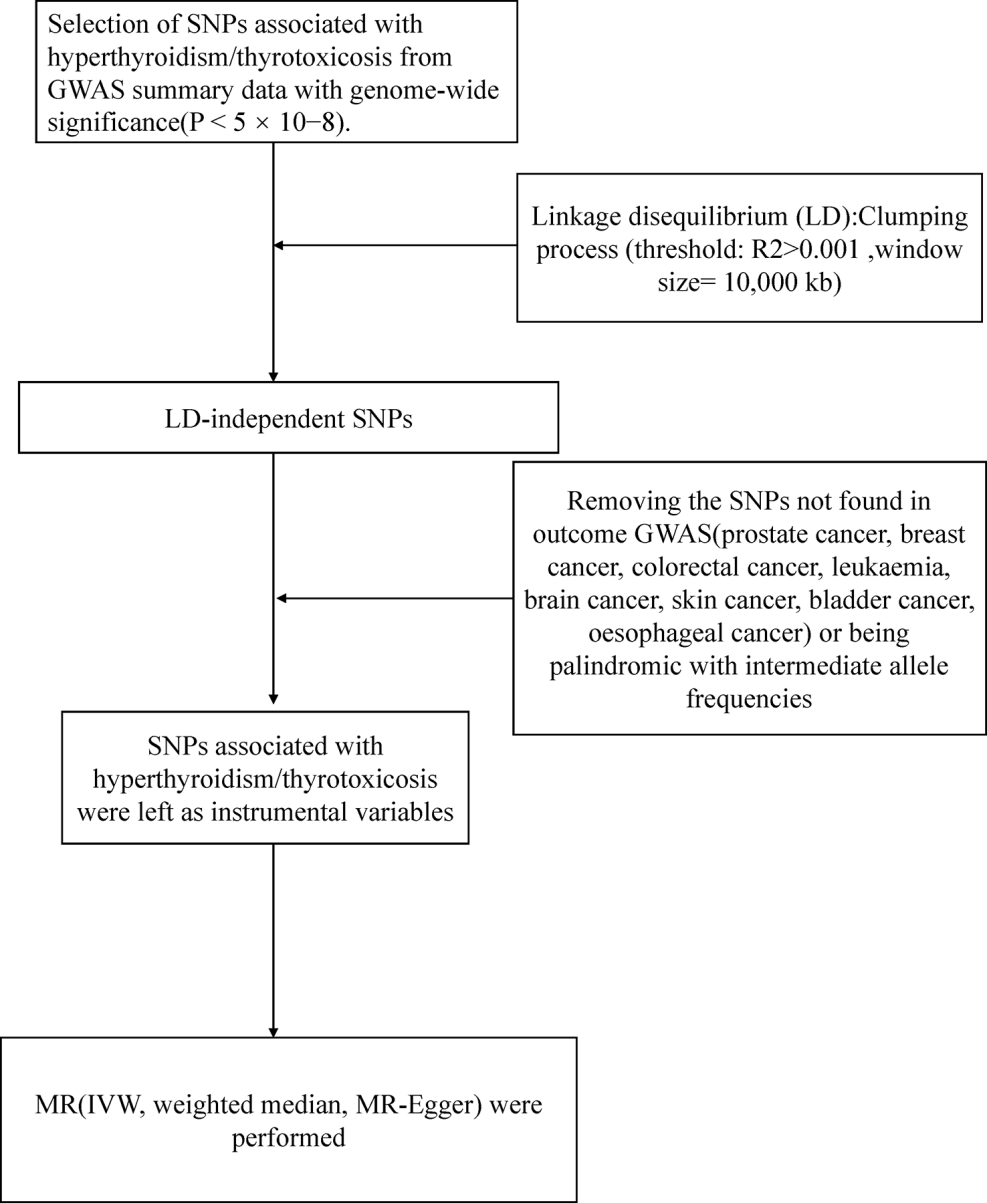
Workflow of Mendelian randomization study revealing causality from hyperthyroidism on nine types of cancer. IVW, inverse variance weighted; MR, Mendelian randomization; SNP, single-nucleotide polymorphisms.

**Figure 1** presented an overview of the study design. We obtained hyperthyroidism exposures from the MRC Integrative Epidemiology Unit (MRC-IEU) consortium with 462933(3545 cases with hyperthyroidism and 459388 controls) individuals of European ancestry (**Table 1**). 13 single-nucleotide polymorphisms (SNPs) significantly associated with hyperthyroidism were identified as instrumental variables that all meted the general genome-wide significance threshold (p < 5 × 10−8, linkage disequilibrium [LD] R2>0.001 within a 10,000 kb window). The detailed information of 13 independent, genome-wide SNPs was listed in **Supplementary Table 1**. The F-statistics was computed to assess weak instrument bias, and they were all larger than the conventional value of 10, showing the strong potential of the instruments to represent hyperthyroidism (16).

**Table 1:** Basic information of population in the study.

Phenotype	GWAS ID	Consortium	Cases (n)	Controls (n)	Population
Exposure					
hyperthyroidism/thyrotoxicosis	ukb-b-20289	MRC-IEU	3545	459388	European
Outcome					European
Prostate cancer	ukb-d-C3_PROSTATE	Neale lab Consortium	6321	354873	European
Lung cancer	ieu-b-4954	UK Biobank	2671	372016	European
Breast cancer	ukb-d-C3_BREAST_3	Neale lab Consortium	9721	351473	European
Colon cancer	ieu-b-4965	UK Biobank	5657	372016	European
Leukemia	ieu-b-4914	UK Biobank	1260	372016	European
Brain cancer	ieu-b-4875	UK Biobank	606	372016	European
Skin cancer	ukb-b-8416	MRC-IEU	3372	459638	European
Bladder cancer	ukb-d-C67	Neale lab Consortium	1554	359640	European
Esophageal cancer	ieu-b-4960	UK Biobank	740	372016	European

MRC-IEU, MRC Integrative Epidemiology Unit.

### 2.2 GWAS summary data for outcome

Large-scale GWAS data were obtained for nine types of cancer (Prostate, Lung, Breast, Colon, Leukemia, Brain, Skin, Bladder, Esophagus) as outcome factors. We respectively obtained data on prostate cancer (6321 cancer cases and 354873 controls), breast cancer (9721 cancer cases and 351473 controls), and bladder cancer (1554 cancer cases 359640 controls) from GWAS meta-analysis from Neale lab Consortium involving European ancestry (**Table 1**). The data for lung cancer (2671 cancer cases and 372016 controls), colorectal cancer (5657 cancer cases and 372016 controls), leukemia (1260 cancer cases and 372016 controls), and brain cancer (606 cancer cases and 372016 controls) was derived from UK Biobank. For skin cancer, we obtained data from the MRC-IEU consortium, consisting of 3372 cancer cases and 459638 controls. This study used only published GWAS data and did not involve individual-level data. All exposure and outcome summary data came from the IEU OpenGWAS project (https://gwas.mrcieu.ac.uk/).

### 2.3 Statistical analyses

Several Mendelian randomization (MR) approaches, including the inverse variance weighted (IVW), weighted median, and MR-Egger, were used to investigate MR estimates of hyperthyroidism for nine cancers after harmonization of effect alleles across the GWASs of hyperthyroidism and nine cancers. Various approaches were performed because they had different fundamental assumptions about horizontal pleiotropy. An IVW meta-analysis of Wald ratios for individual SNPs was considered the primary outcome, assuming that the instruments could influence the outcome only by interest exposure and not through any alternative pathway (13). We additionally used MR-Egger and weighted median methods to complete IVW estimates since these methods provided more reliable estimates in a broader range of scenarios but could have been more efficient.

Sensitivity analysis was performed to explore underlying pleiotropy and the heterogeneity in the MR study. We represented potential horizontal pleiotropy with heterogeneity markers from the IVW approach (Cochran Q-derived P < 0.05). The MR-Egger regression was carried out to calculate the intercept indicating directional pleiotropy (P-value<0.05 was considered to be the existence of directional multiplicity), and the values greater than or less than zero indicated that the IVW estimate may be biased. Association estimates for SNP exposure and SNP outcome were involved in MR-Egger. We estimated the causal effect of exposure on outcomes with the weighted regression line slope independent of horizontal pleiotropy. The leave-one-out method was performed to assess the sensitivity of the results by sequentially removing one SNPs at a time to know whether the MR estimate was driven or biased by a single SNP.

The MR analysis was implemented by the package TwoSampleMR (version 0.4.25) in R (version 4.2.1).

## 3 Results

### 3.1 Instrumental variables selection

13 hyperthyroidism-related SNPs were identified, and the SNP information, including the name, chromosome location, effect allele (EA), other alleles, and effect allele frequency (EAF), was listed in **Supplementary Table 1**.

### 3.2 Causal effect from hyperthyroidism to nine type cancers

**Figure 2:**
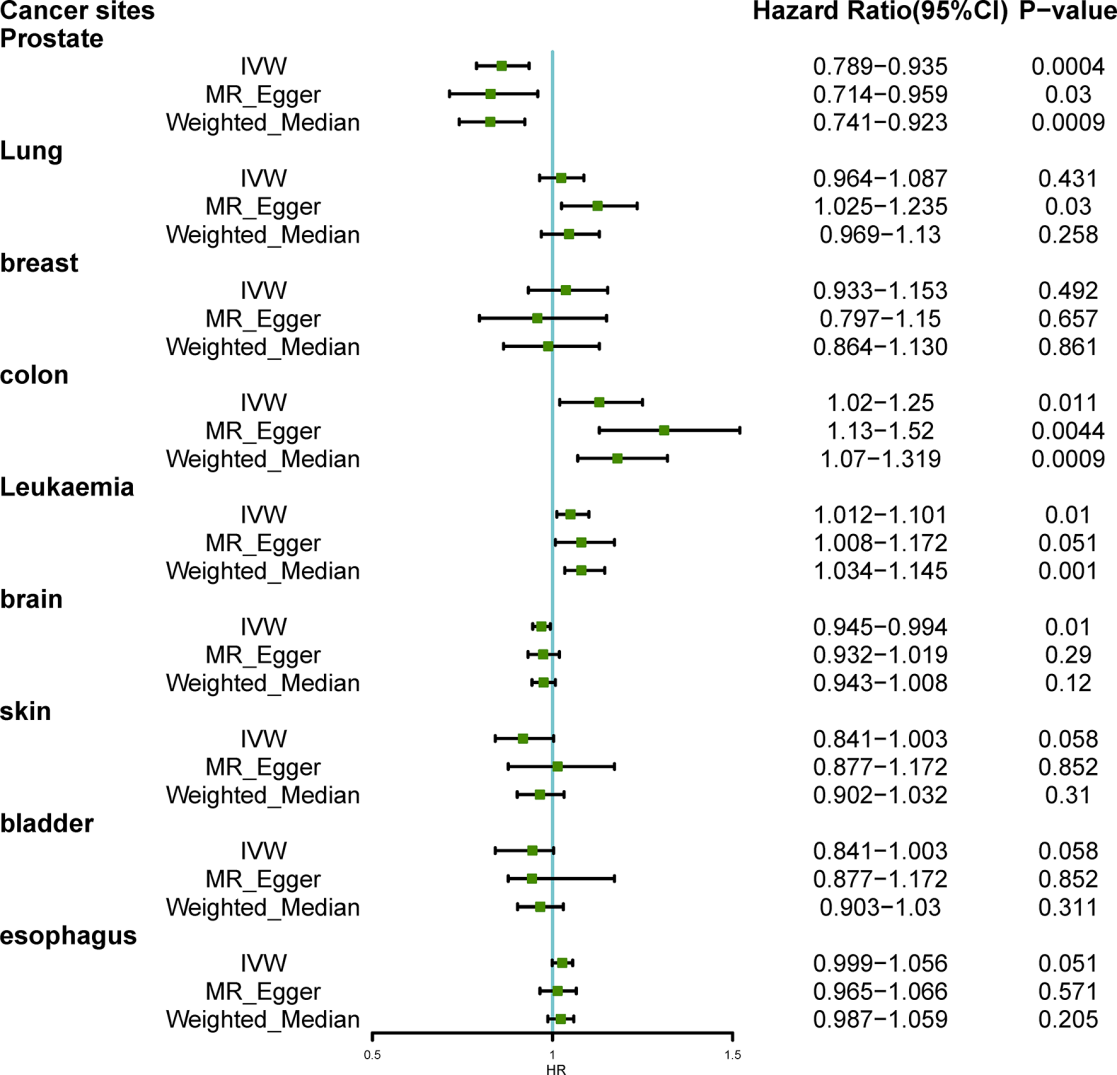
Hazard Ratio plot for hyperthyroidism and nine types of cancer; IVW, inverse variance weighted.

**Figure 2** showed the results of estimated causal associations between hyperthyroidism and nine types of cancers. We found hyperthyroidism was associated with a declining risk of occurrence of prostate cancer (odds ratio (OR)_IVW_= 0.859, P= 0.0004; OR _MR-Egger_=0.828, P= 0.03; OR _weighted median_= 0.827, P=0.0009). Additionally, there was a significant association between genetically predicted hyperthyroidism and colon cancer (OR _IVW_= 1.13, P= 0.011; OR _MR-Egger_= 1.31, P= 0.004; OR _weighted median_= 1.18, P= 0.0009). The method of MR-Egger showed that hyperthyroidism was related to an increased risk of lung cancer (OR= 1.125, P= 0.03), while it was not significant in IVW and weighted median (All P>0.05). An increasing risk from hyperthyroidism to leukemia was found based on the result of IVW, and weighted median (OR _IVW_= 1.05, P= 0.01; OR _weighted median_= 1.08, P= 0.001), and MR-Egger failed to obtain a significant result (P= 0.051). The single SNP effect and the combined effects of each MR method were represented with the scatter plots (**Supplementary Figure 1**). We did not observe causal associations between hyperthyroidism and cancers of the breast, brain, skin, bladder, and esophagus (all P>0.05).

### 3.3 Sensitivity analysis

We carried out pleiotropy, heterogeneity, and sensitivity analysis to further examine the reliability of the results. There was no statistical heterogeneity in the MR study with the method of MR-Egger and IVW (All P >0.05). Furthermore, no horizontal pleiotropy was found in the MR-Egger intercept tests (**Supplementary Figure 2**). The leave-one-out sensitivity analysis showed that the genetic prediction of estimating the causal effects between hyperthyroidism and nine cancers was robust (**Supplementary Table 2**).

## 4 Discussion

In the current study, a two-sample MR analysis using instrumental variables of large-scale GWAS was performed to evaluate the causal relationship between hyperthyroidism and nine types of cancers using genetic data from populations of European ancestry. We found a negative causal association between hyperthyroidism and prostate cancer. Simultaneously, hyperthyroidism is associated with increased colon cancer and leukemia incidence. Results from a two-sample MR analysis suggested that hyperthyroidism was unrelated to the risk of lung cancer, breast cancer, brain cancer, skin cancer, bladder cancer, and esophageal cancer.

Previous studies on the association between hyperthyroidism and colon cancer have yielded inconsistent results. In one of the reviewed studies, the association between thyroid dysfunction and cancer risks was analyzed, revealing an increased risk of thyroid, breast, and prostate cancers in individuals with hyperthyroidism. This finding suggests that excessive amounts of thyroid hormones may stimulate cancer cell proliferation in certain tissues (17). A nested case-control study conducted by Boursi et al. found that hyperthyroidism was associated with a modest increase risk of colon cancer (adjusted OR = 1.21). However, they also detected a similar risk in untreated hypothyroidism (adjusted OR = 1.16) (18). Abby L’Heureux et al., in a population-based case–control study, revealed the negative causal association between hyperthyroidism and colon cancer (adjusted OR 0.74) (19). The main reason for the discrepancy between the above results might be the heterogeneity of the study population. Our results in the two-sample MR analysis support a causal relationship between hyperthyroidism and a high risk of colon cancer. Our study population was mainly a European sample, which was consistent with the study’s main population by Boursi et al. But at the same time, it was worth noting that the application of our findings might be limited. In line with our MR results, previous studies had presented a positive relationship between hyperthyroidism and leukemia. For instance, Ghalaut et al. found that the values of FT3, FT4, T3, and T4 in patients with acute leukemia were significantly higher than those without. Moreover, lower TSH levels were observed in acute leukemia patients compared to controls.

An interesting finding in this study was that hyperthyroidism had a negative association with the risk of prostate cancer. This result was inconsistent with previous results. A community-dwelling population based on Busselton Health Survey showed a higher level of FT4 was associated with an increased risk of prostate cancer (20). Likewise, a prospective study by Alison M. Mondul et al. found a decreased risk of prostate cancer among men with a hypothyroid state (21). On the contrary, some hypotheses, such as the protective effect of thyroid hormone on cancer, have also been proposed (2, 22). For example, the TRα and TRβ genes encoded thyroid hormone receptors (TRs), which are ligand-dependent transcription factors in the nuclear receptor superfamily (22). There was growing evidence that these receptors also inhibited cell transformation and acted as tumor suppressors, apart from their roles in growth, development, and metabolism. Various tumors like lung and breast cancer were reported to associate with hormone receptor inactivation mutations, thereby preventing wild-type TRs from entering the target gene and producing hypothyroidism. The possible explanation behind the differences in the results of the association between hyperthyroidism and prostate cancer might be residual confounding or reverse causality in observational retrospective studies or a lack of strength in the MR study due to the relatively low number of prostate cancer cases. Need to be a more rigorous prospective randomized controlled study in the future to provide higher quality evidence to confirm these results.

There was a growing body of evidence suggesting that thyroid hormones played important roles in regulating cellular processes in various tissues and organs, including those relevant to cancer development and progression. The mechanisms underlying the effects of thyroid hormones on cancer cells have been shown to be multifaceted and complex, involving both nuclear hormone receptors and cell surface receptors (23). Studies suggested that thyroid hormones’ effects on tumor cells occur *via* nuclear hormone receptors and cell surface receptors on integrin αvβ3. In breast, brain, liver, thyroid, and colon cancers, thyroid hormones activated intracellular signaling pathways that promote cancer progression. Moreover, local levels of thyroid hormones were regulated *via* activation and deactivation of iodothyronine deiodinases in various organs, which affects cancer progression and development (24). Furthermore, recent research had also identified cuproptosis, a copper-dependent regulated cell death mechanism, as a potentially new treatment strategy for cancer. A lncRNA signature linked to cuproptosis was found to be associated with prognostic predictors for stomach adenocarcinoma (STAD), indicating its potential as a biomarker for risk stratification, evaluation of possible immunotherapy, and assessment of treatment sensitivity for STAD (25, 26). Although thyroid hormones played an important role in the processes related to cancer occurrence and progression, their mechanisms were complex and multifaceted. Further research was needed to better understand these mechanisms and developed targeted interventions based on thyroid hormone functional modulators. The results of these studies might provide guidance for the development of potential therapeutic strategies for individuals at risk of thyroid dysfunction and cancer.

Our MR results did not support the causal relationship between hyperthyroidism and the risk of lung cancer, breast cancer, brain cancer, skin cancer, bladder cancer, and esophageal cancer. It was noteworthy that the MR analysis obtained a positive association between hyperthyroidism and lung cancer from the method of MR-Egger, but not in IVW and weighted median. Considering the lack of statistical strength of MR analysis, we did not consider this association to be statistically significant (13, 16). A nationwide cohort study by Mette Søgaard et al. revealed that women with hyperthyroidism had a higher risk of breast cancer (27). Simultaneously, a large prospective population study reported that a low level of thyrotropin was related to an increased incidence of lung cancer. In addition, this association was more prominent after two years of follow-up (28). Turkyilmaz et al. prospectively found hyperthyroidism was linked to an increased incidence of esophageal cancer (29). There have been no observational studies on the relationship between hyperthyroidism and cancers of the brain, skin, and bladder up to now. The discrepancy between the observational study and our MR analysis might result from the inconsistency of the statistical methods and the subject population of the study.

This was the first MR study to assess the association between hyperthyroidism and nine site-specific cancers comprehensively. The main advantage of our research was the two-sample MR study design, which could reduce unobserved confounding and reverse causality and might lead to inconsistent results with observational studies (12, 30, 31). Furthermore, since our research uses only data from European populations, the results are likely unaffected by demographic stratification. But at the same time, it also limited the extension to other populations. However, several limitations of this study should be noted. A major limitation was that the number of cancer cases in the MR analysis was relatively less compared with controls, resulting in a relatively low estimation accuracy, which might lead to false negative results in this study. Then, we failed to conduct analysis at the patient level, such as hierarchical analysis of age, sex, race, and other information owing to the lack of original data (32, 33). In addition, horizontal pleiotropy was a common and challenging problem in MR analysis. However, we conducted sensitivity analyses like the leave-one-out method and MR-Egger and found no detectable directional pleiotropy. It showed that our results were not like to be affected by pleiotropy, but the possibility of bias cannot be ruled out. Last but not least, our study suggested a potential causal association between hyperthyroidism and types of cancer. Still, this analysis did not provide evidence for the specific mechanism of tumorigenesis due to the limitations of available data.

## 5 Conclusion

In summary, this two-sample MR study provided evidence of a positive causal association between hyperthyroidism, colon cancer, and leukemia. Simultaneously, it also suggested a negative causal relationship between hyperthyroidism and prostate cancer. The recommended treatment for subclinical and diagnosed hyperthyroidism might be a preventive strategy for colon cancer and leukemia. Moreover, further MR studies might be needed to evaluate the future relationship between hyperthyroidism and essential cancer risk factors.

## Data availability statement

The original contributions presented in the study are included in the article/**Supplementary Material**. Further inquiries can be directed to the corresponding author.

## Author contributions

FX and ZC contributed to the study’s conception and design. All authors contributed to the article and approved the submitted version.
