# Supplementary figures and images for: Causal associations of hyperthyroidism with prostate cancer, colon cancer, and leukemia: a Mendelian randomization study
Source: Front Endocrinol (Lausanne). 2023 May 18;14:1162224. doi: 10.3389/fendo.2023.1162224 (PMC10233060; doi:10.3389/fendo.2023.1162224)

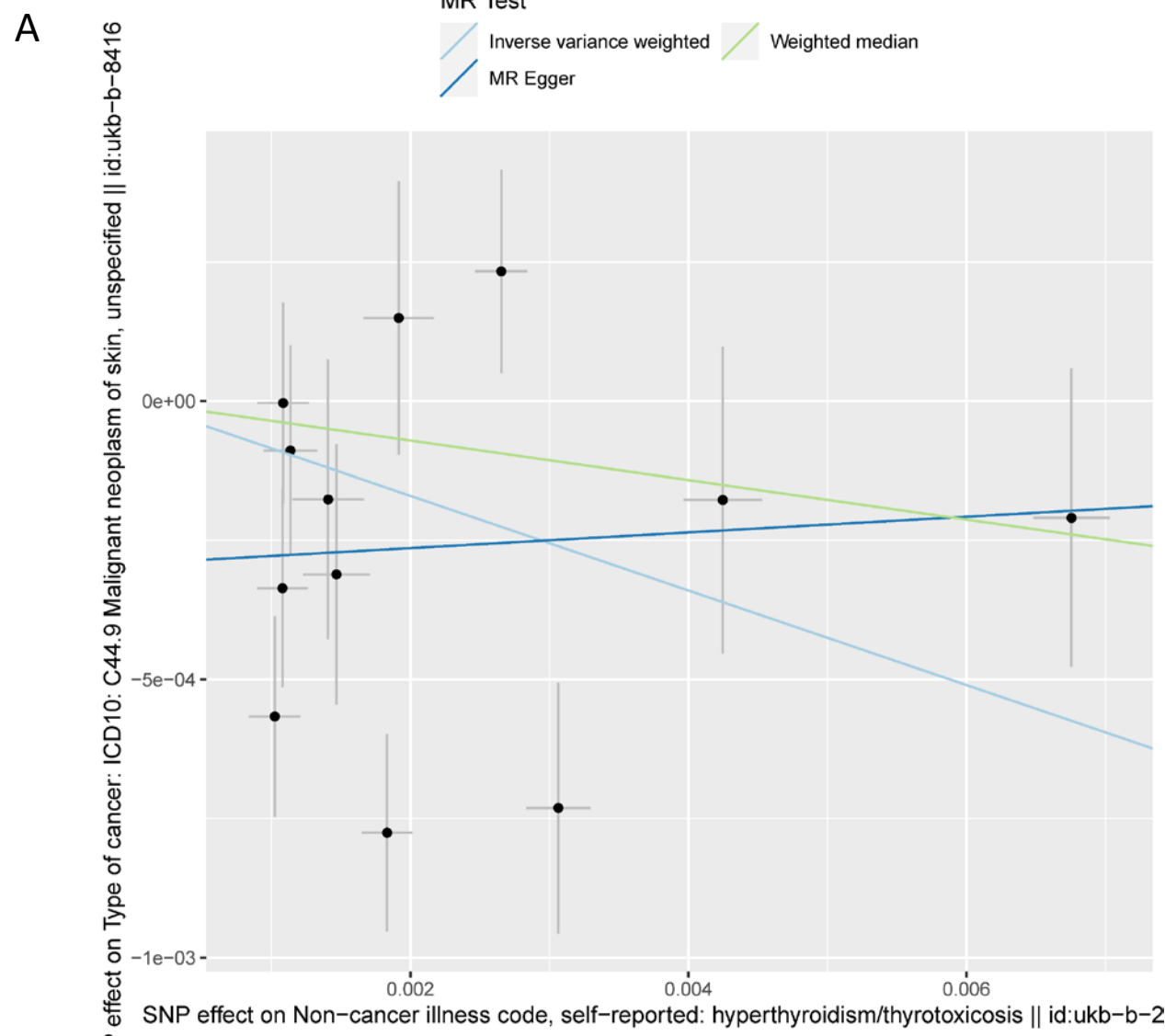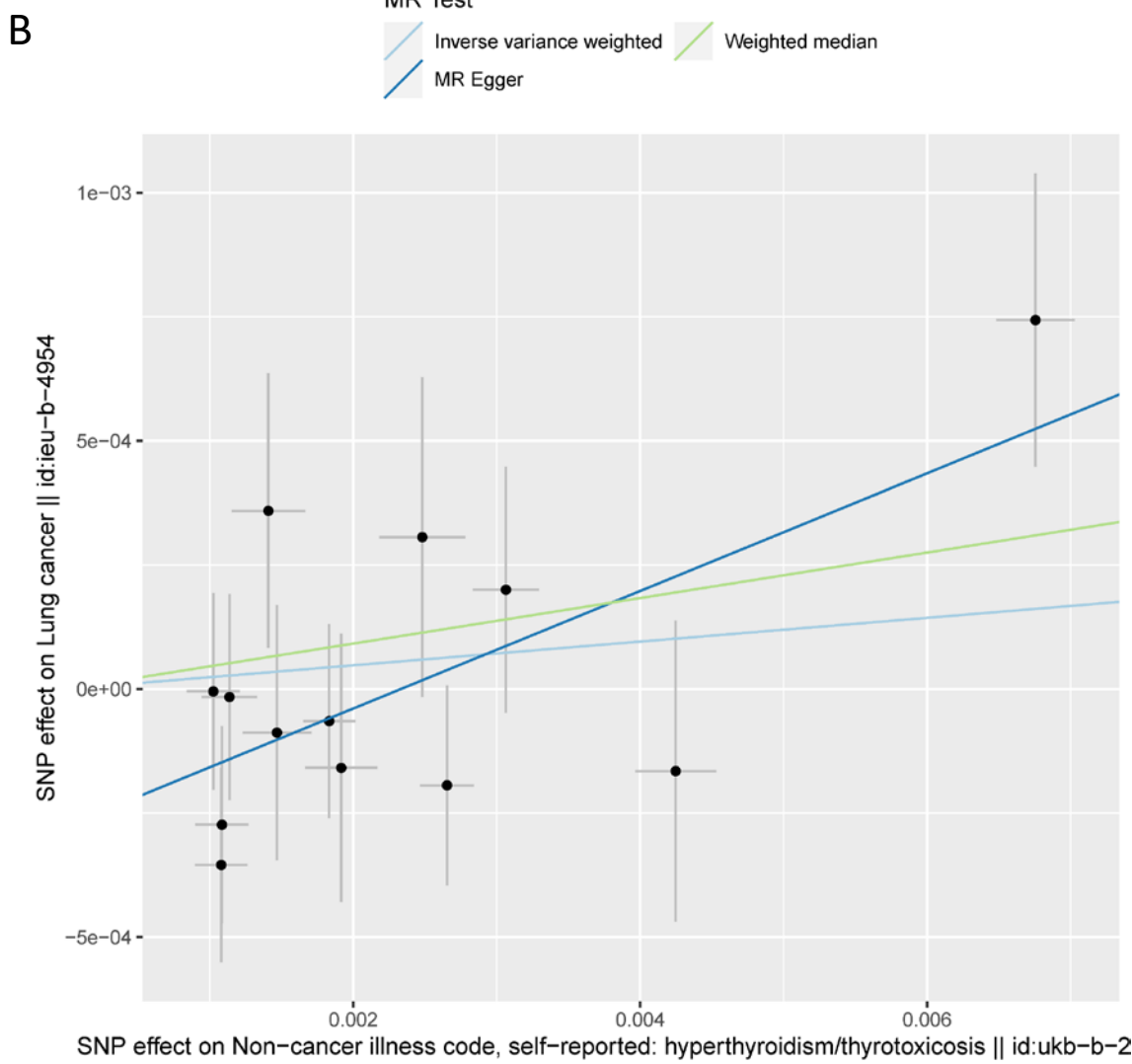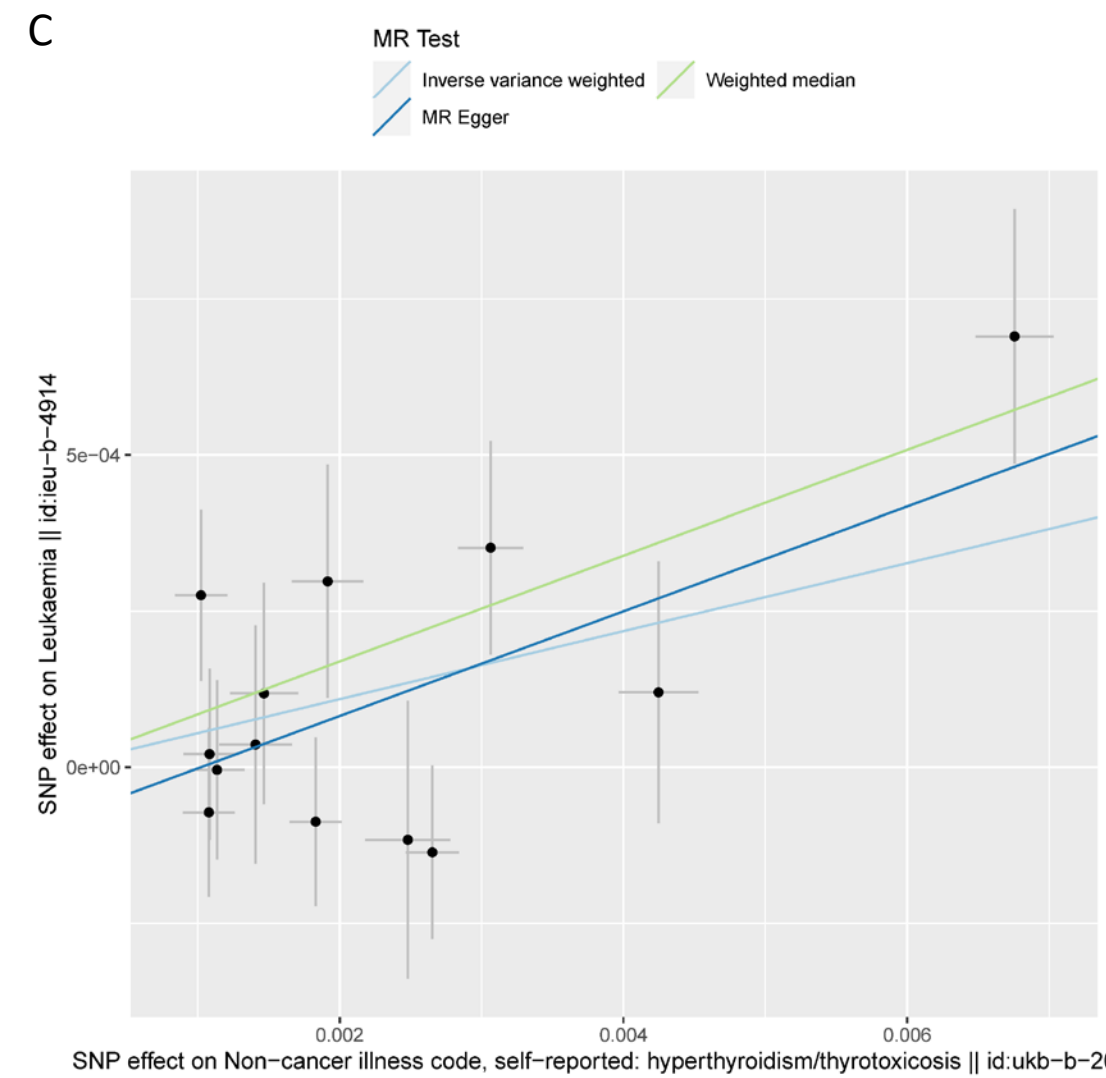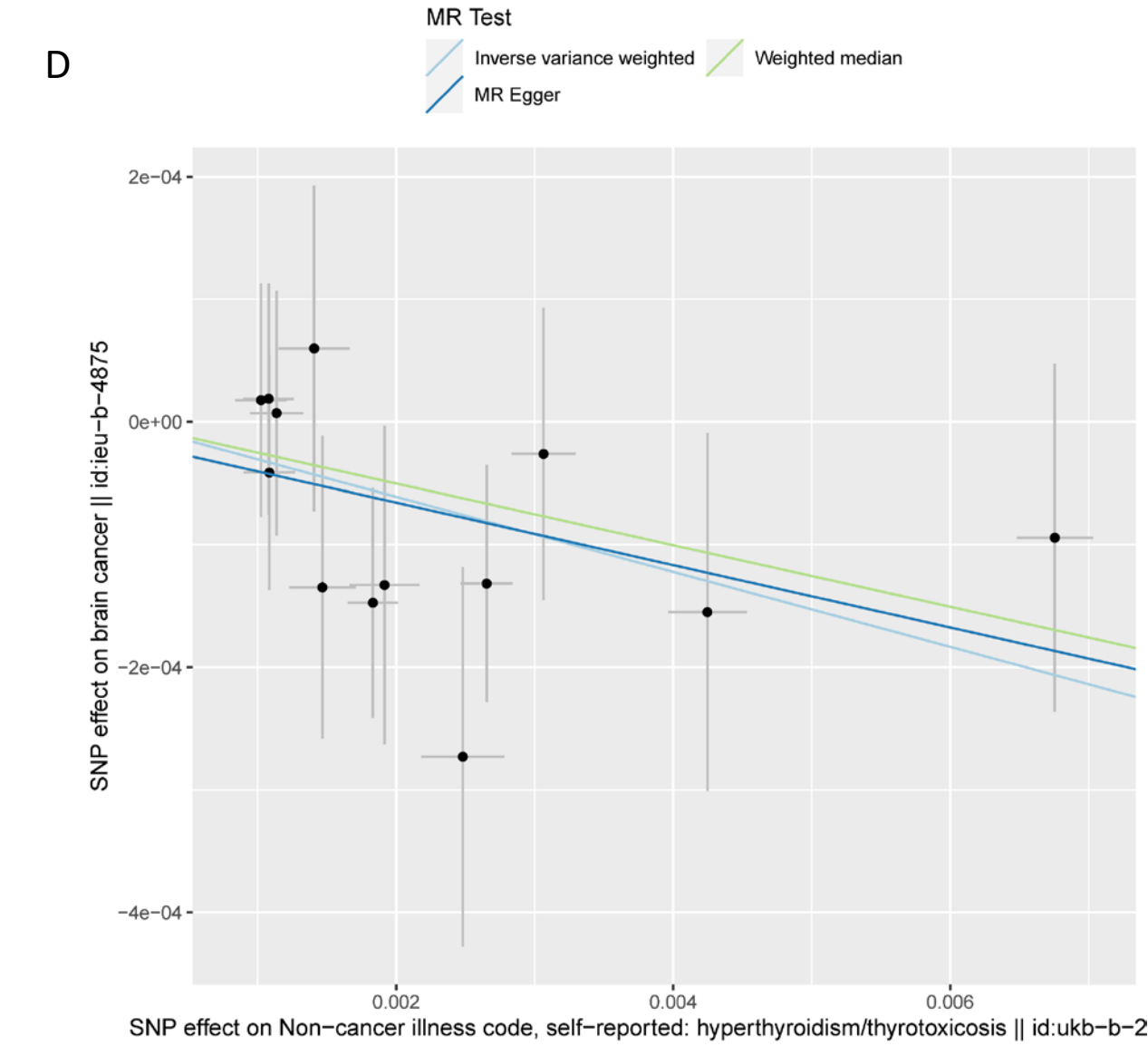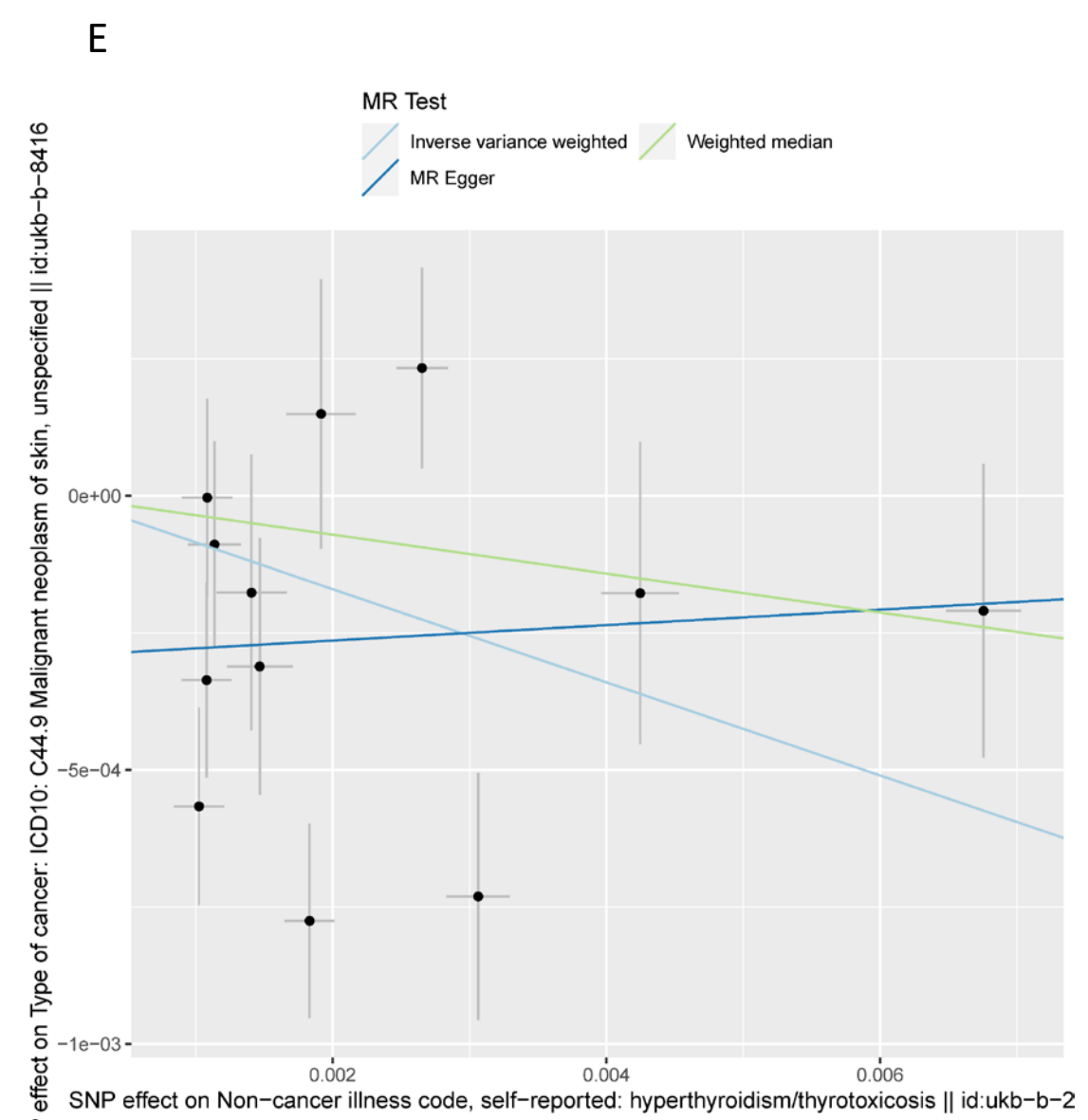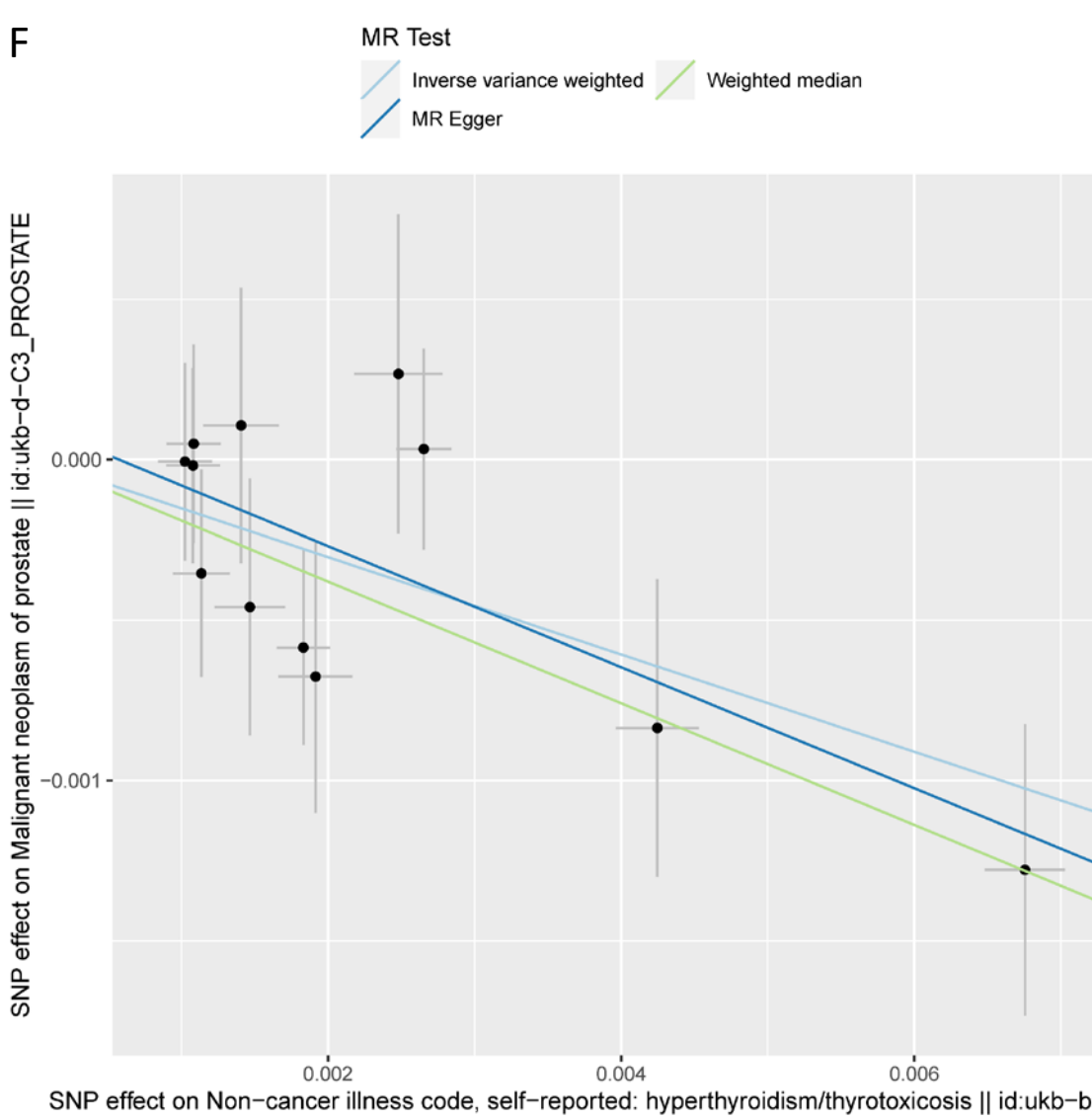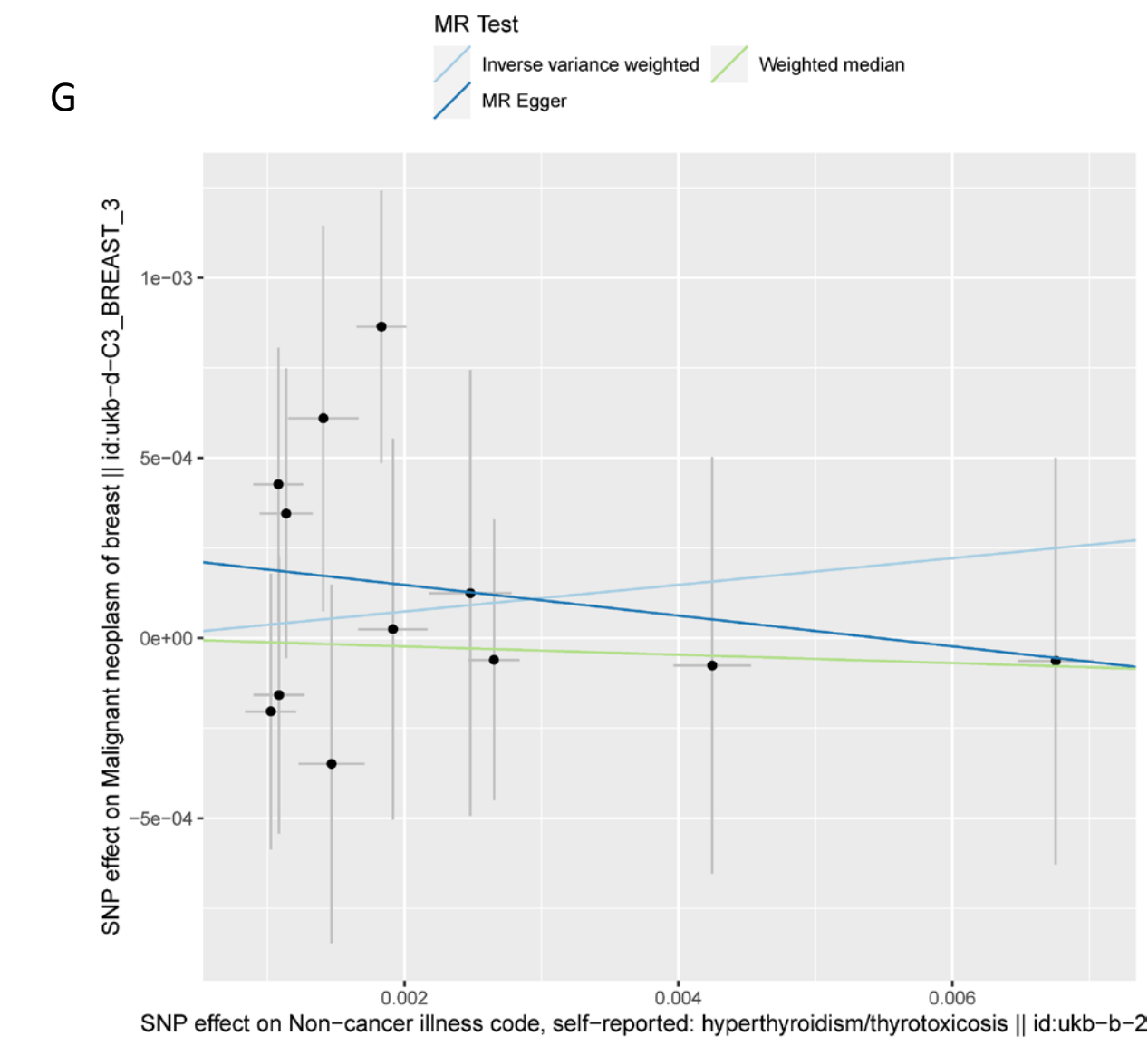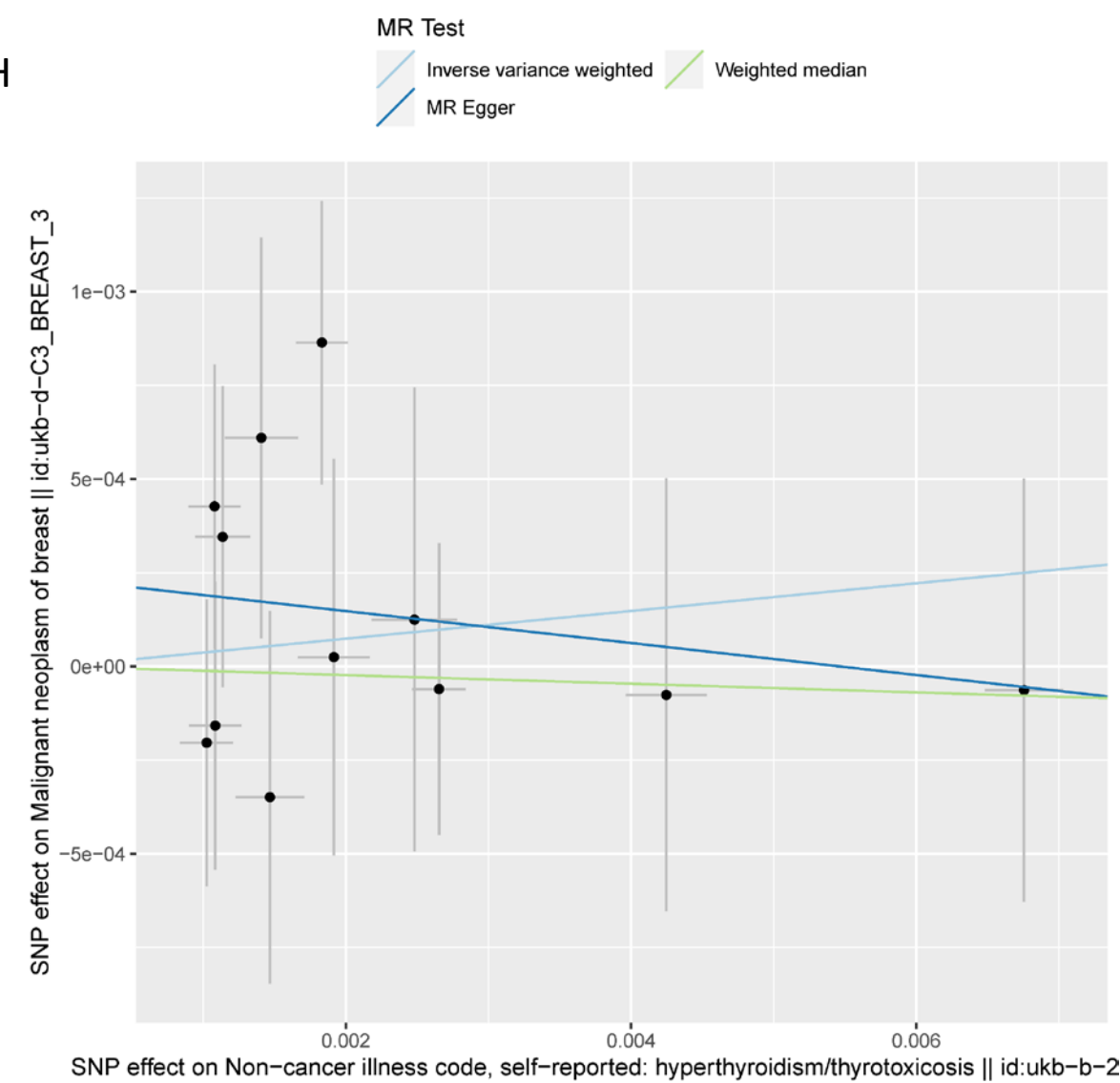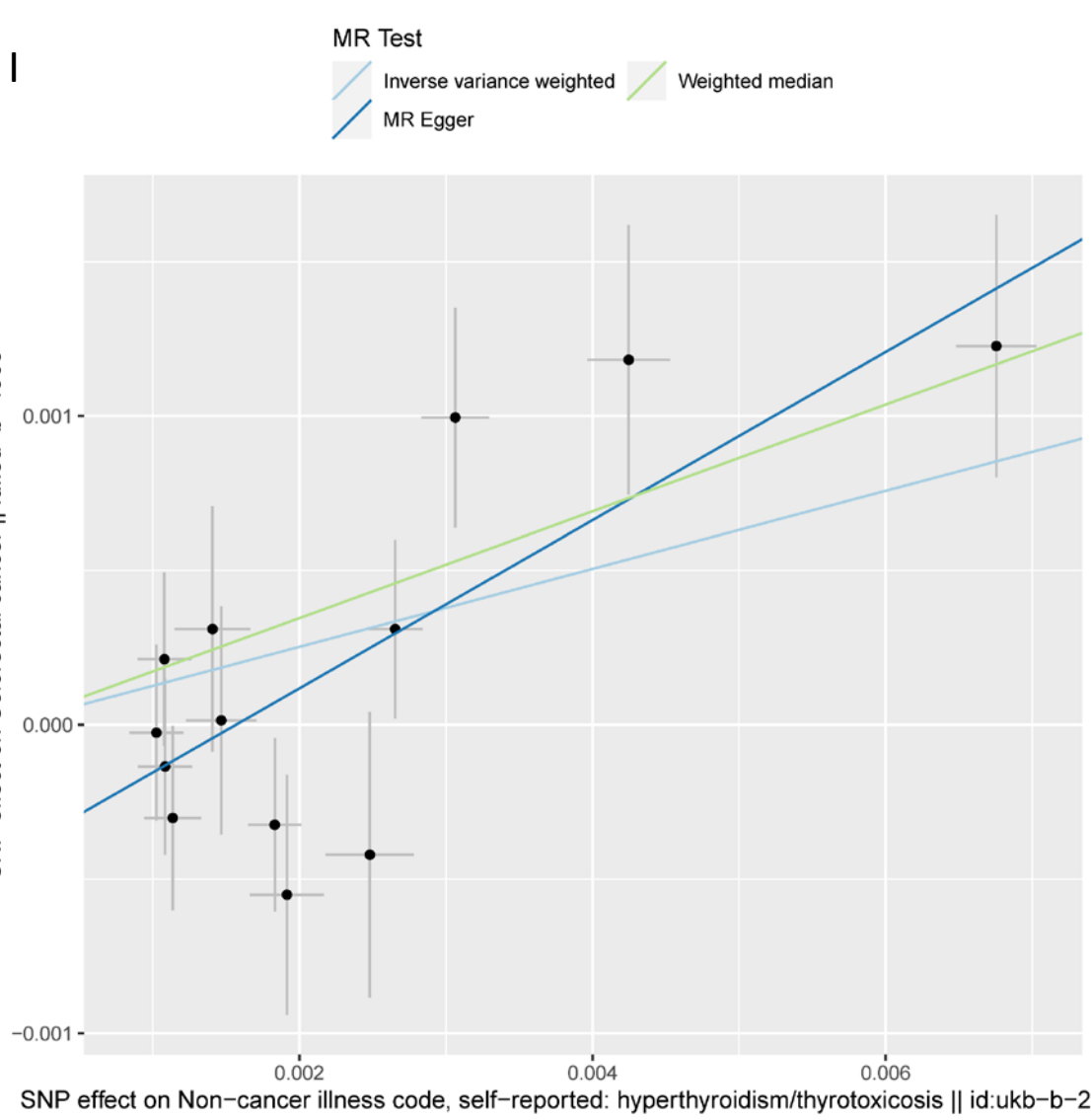

Supplement: Supplementary file 1 [file Image_1.pdf]

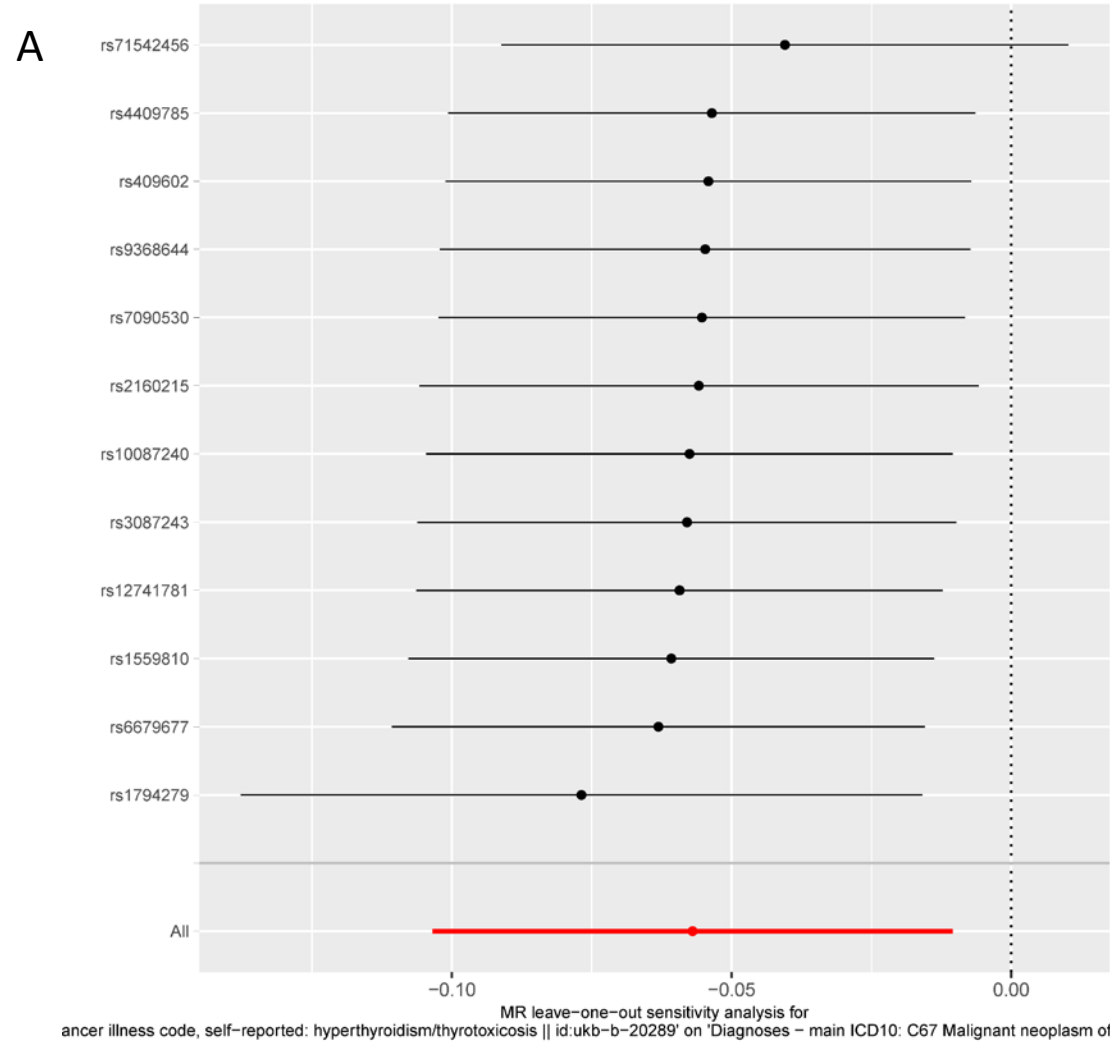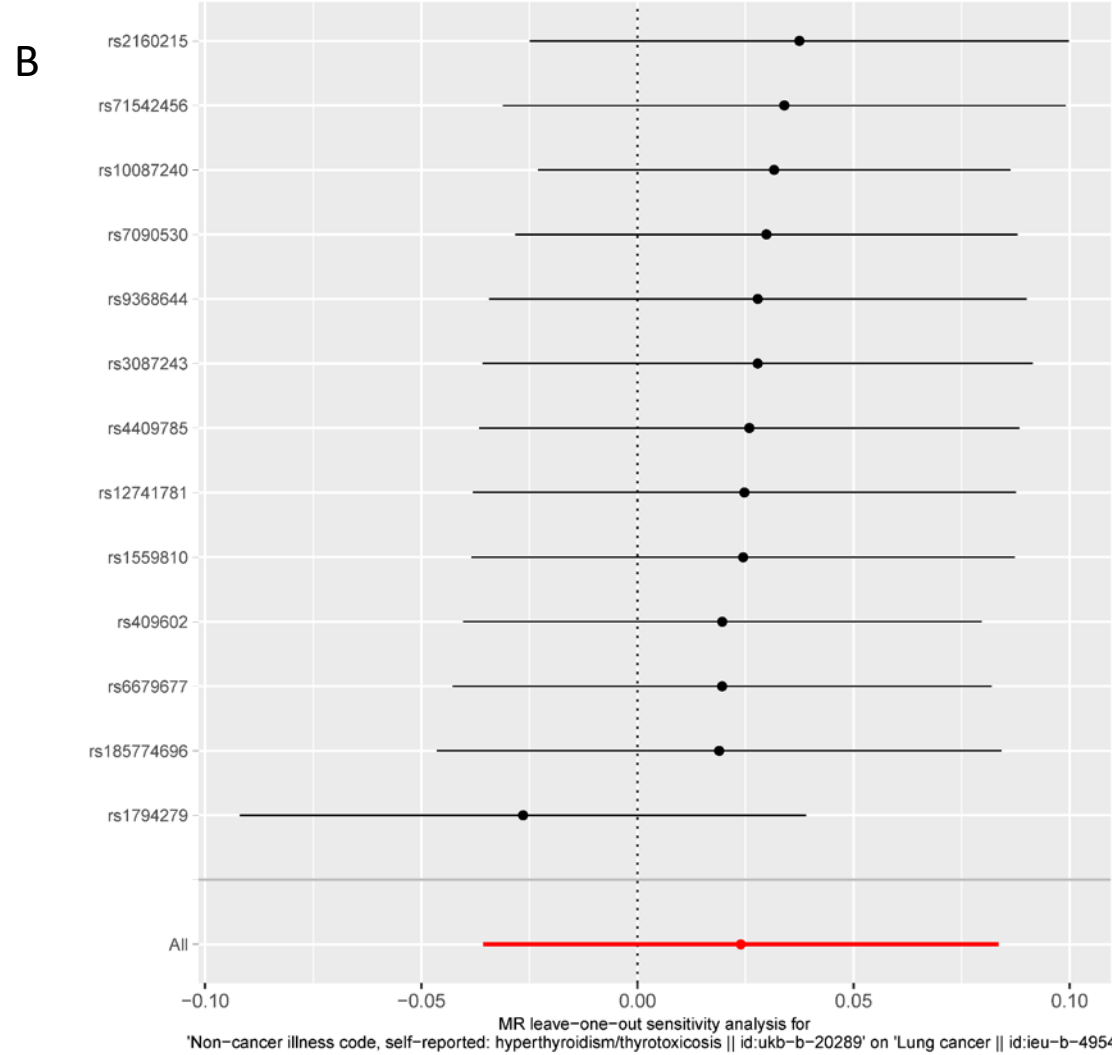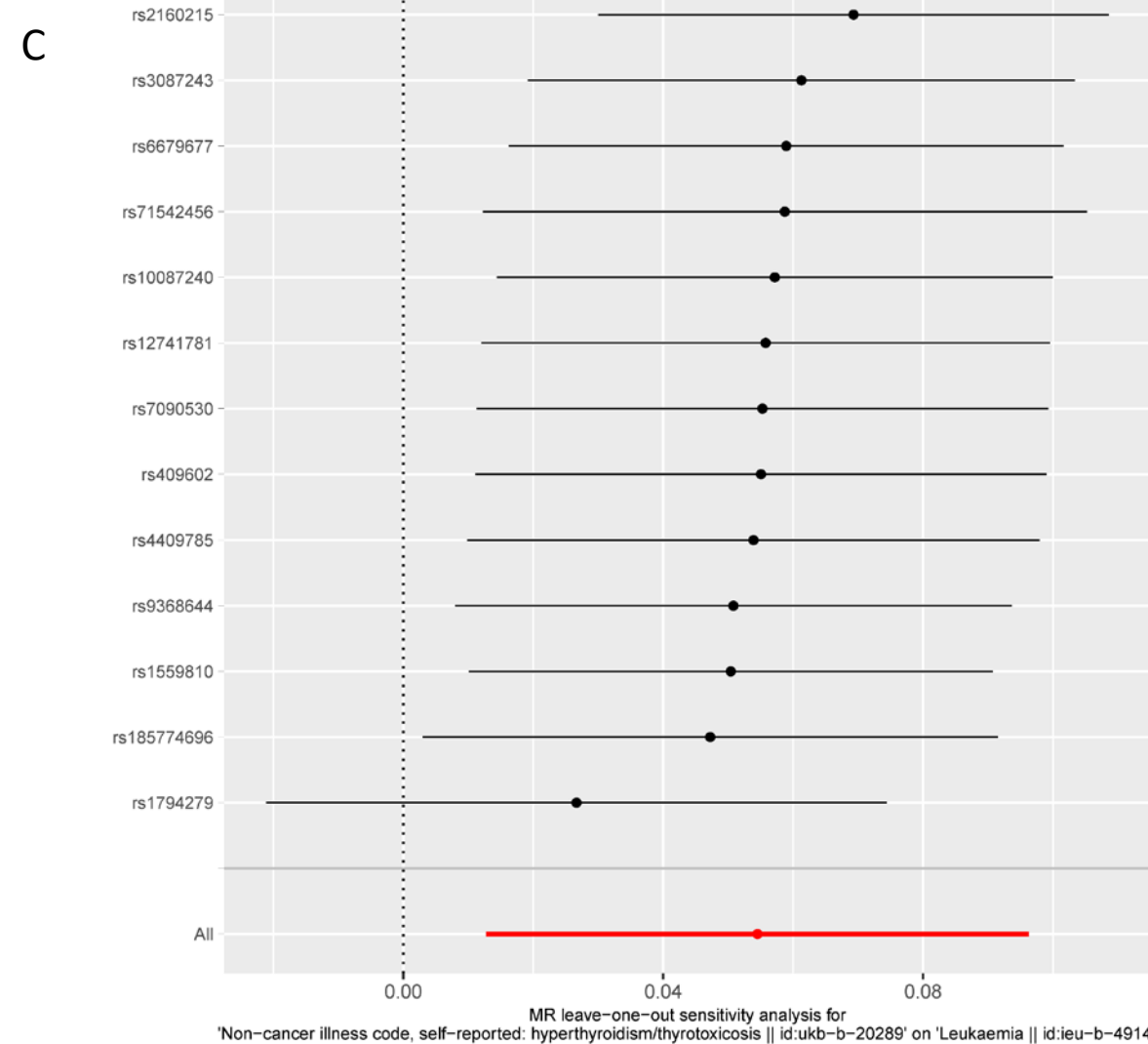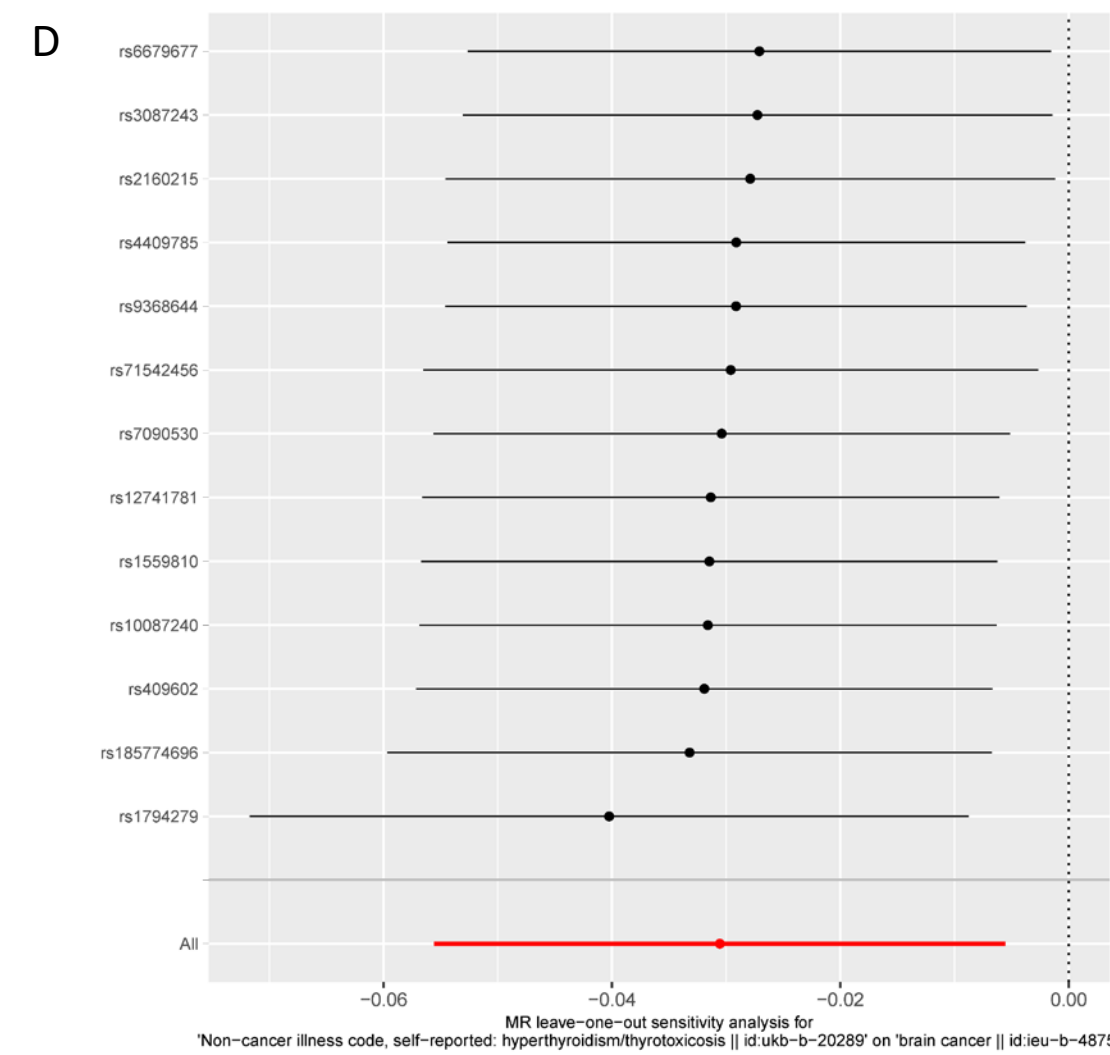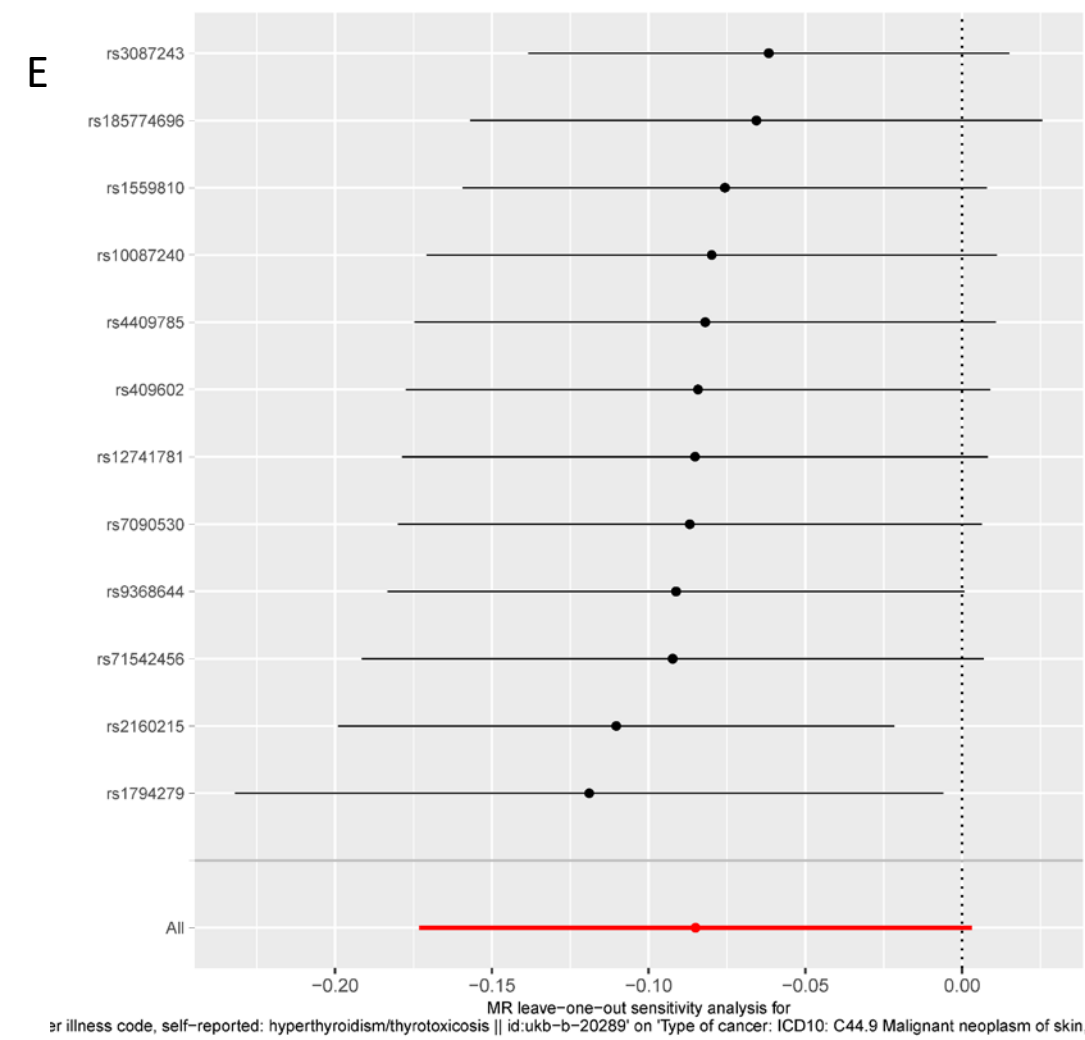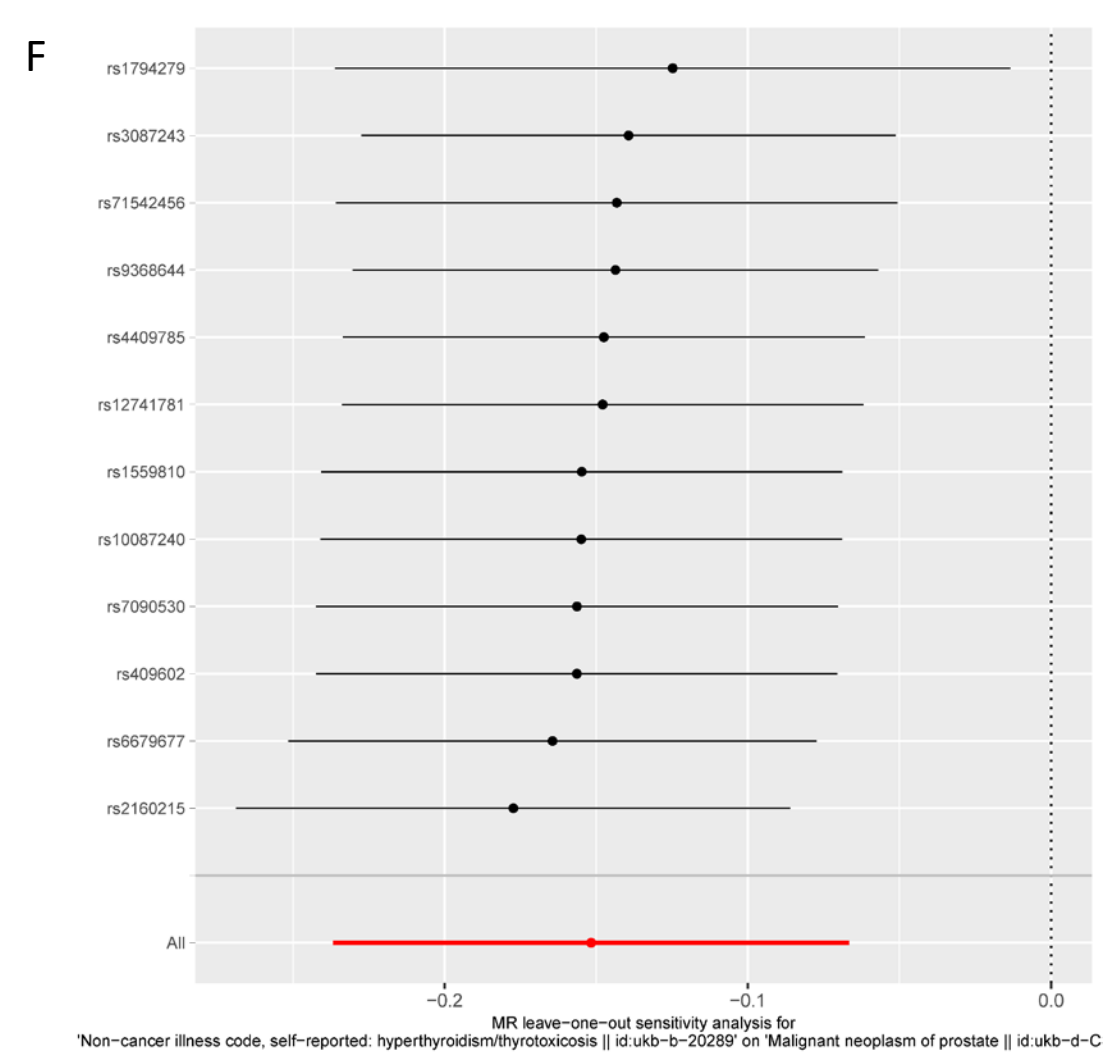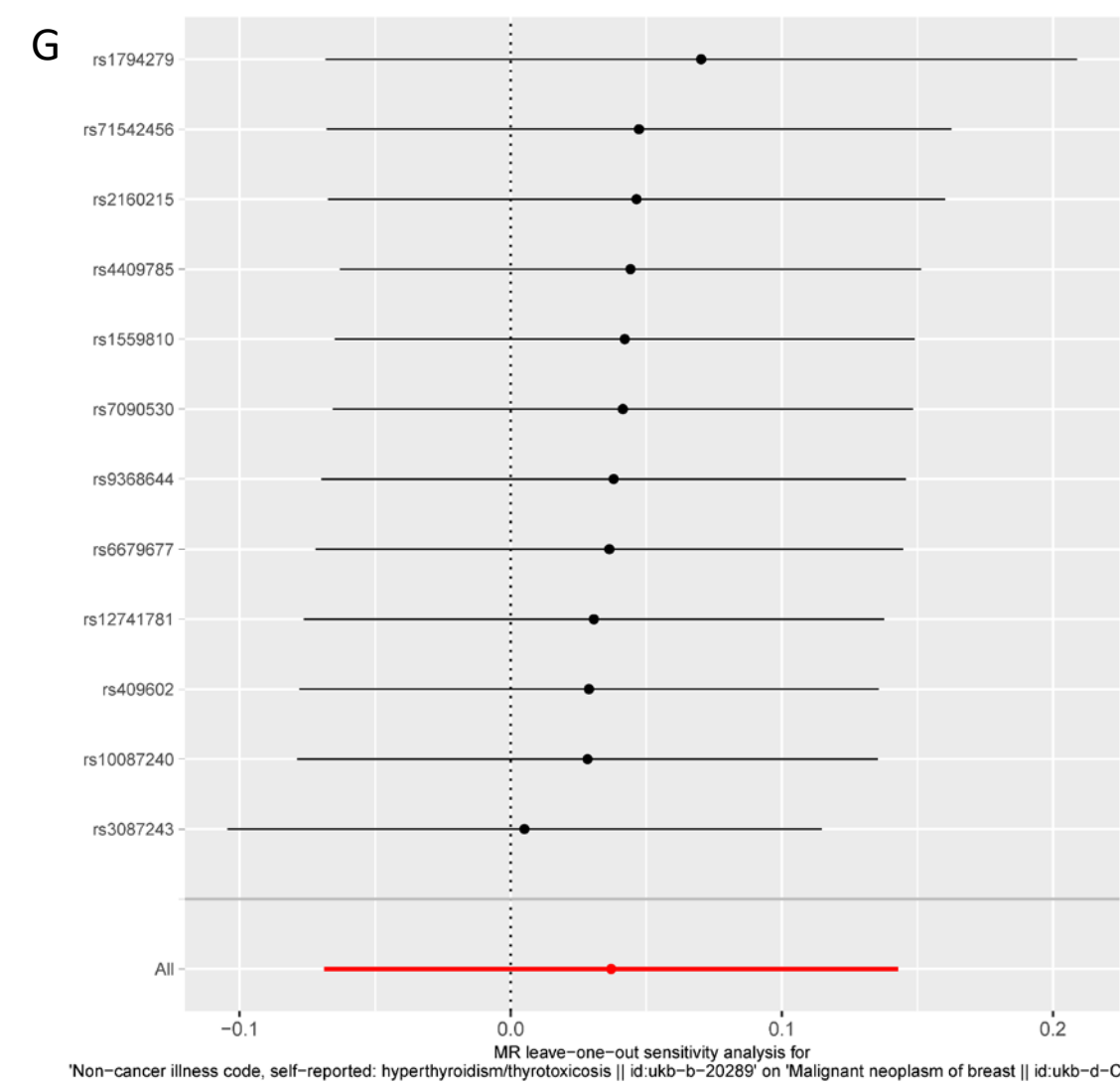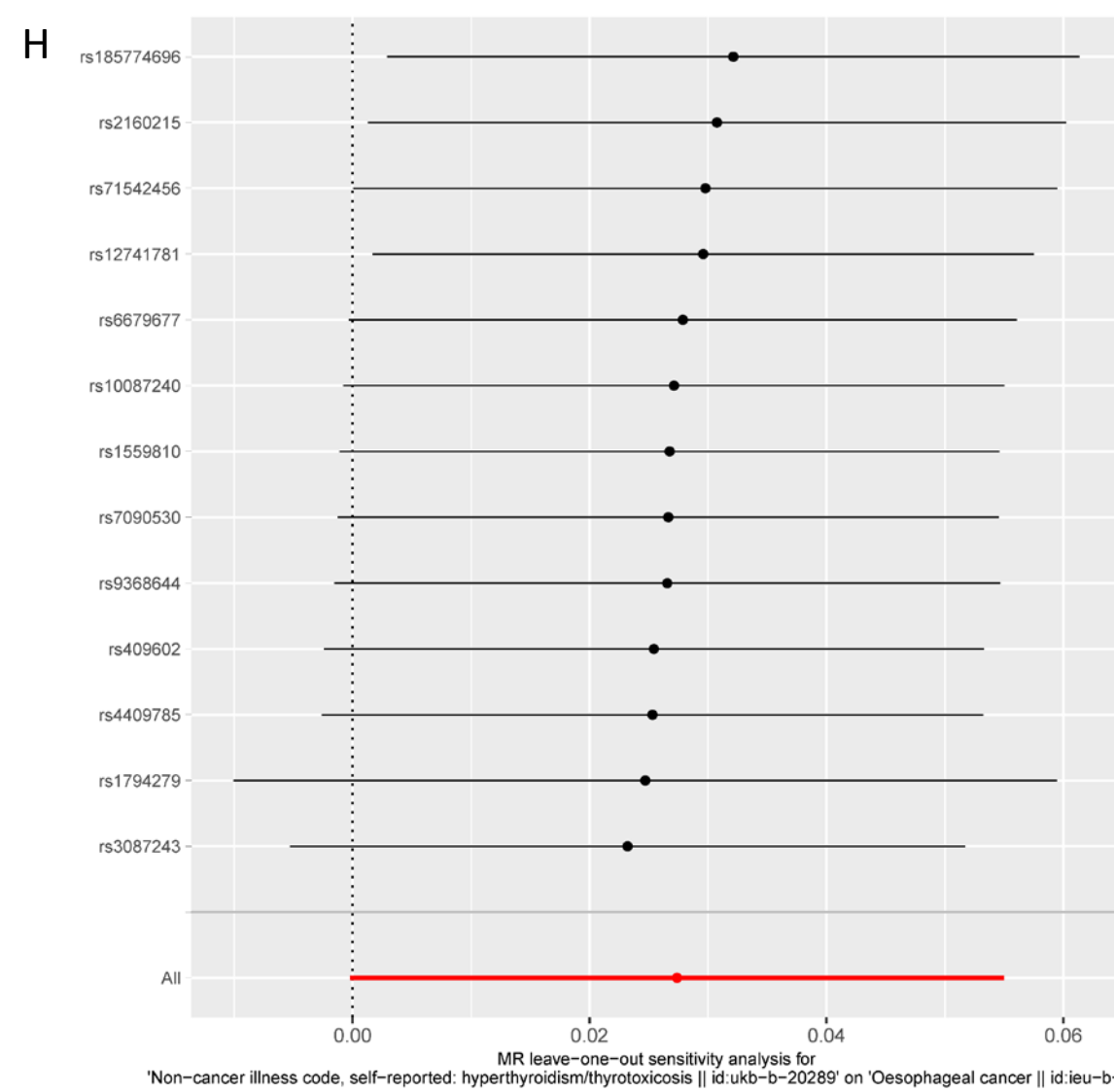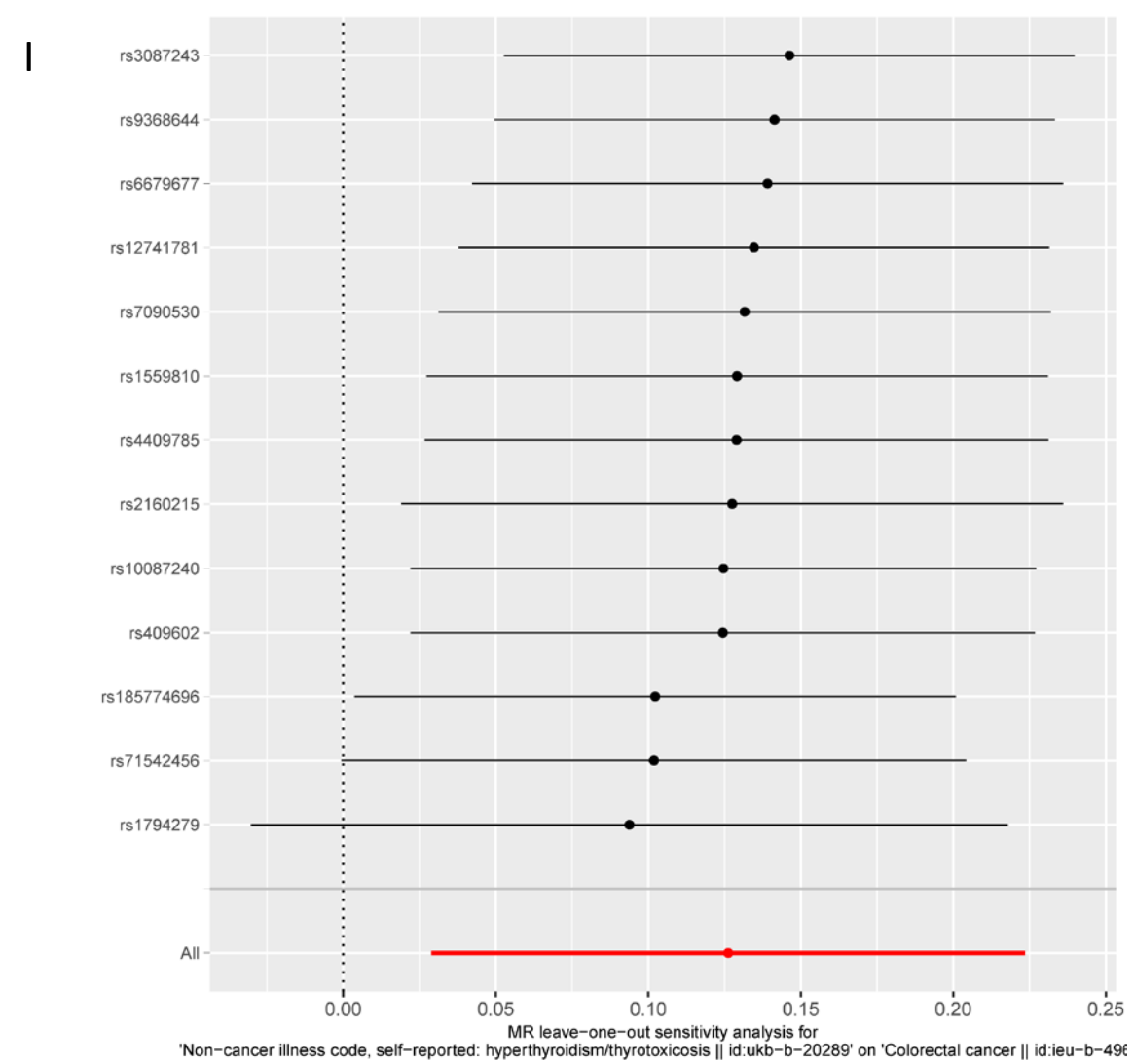

Supplement: Supplementary file 2 [file Image_2.pdf]
